# First Principles Study on the Electronic Structure and Interface Stability of Hybrid Silicene/Fluorosilicene Nanoribbons

**DOI:** 10.1038/srep15734

**Published:** 2015-10-26

**Authors:** Q. G. Jiang, J. F. Zhang, Z. M. Ao, Y. P. Wu

**Affiliations:** 1 College of Mechanics and Materials, Hohai University, Nanjing 210098, China; 2 Centre for Clean Energy Technology, School of Mathematical and Physical Sciences, University of Technology Sydney, PO Box 123, Broadway, Sydney, NSW 2007, Australia

## Abstract

The interface stability of hybrid silicene/fluorosilicene nanoribbons (SFNRs) has been investigated by using density functional theory calculations, where fluorosilicene is the fully fluorinated silicene. It is found that the diffusion of F atoms at the zigzag and armchair interfaces of SFNRs is endothermic, and the corresponding minimum energy barriers are respectively 1.66 and 1.56 eV, which are remarkably higher than the minimum diffusion energy barrier of one F atom and two F atoms on pristine silicene 1.00 and 1.29 eV, respectively. Therefore, the thermal stability of SFNRs can be significantly enhanced by increasing the F diffusion barriers through silicene/fluorosilicene interface engineering. In addition, the electronic and magnetic properties of SFNRs are also investigated. It is found that the armchair SFNRs are nonmagnetic semiconductors, and the band gap of armchair SFNRs presents oscillatory behavior when the width of silicene part changing. For the zigzag SFNRs, the antiferromagnetic semiconducting state is the most stable one. This work provides fundamental insights for the applications of SFNRs in electronic devices.

Two dimensional silicene has recently been synthesized on Ag^1^,^2^,^3^, Ir^4^, Au^5^, and ZrB_2_^6^ substrates and attracted enormous interests to explore its potential applications for electronic devices, because, similar to graphene, it has unique physical and electronic properties, such as it has linear band structure near the Dirac point. However, several issues have restricted the development of silicene electronics, especially the absence of band gap in the electronic structure of silicene^7^,^8^,^9^. It has been reported that fluorination of silicene is an effective method to tune the band gap of silicene^10^. However, the fully fluorinated silicene is a wide-gap insulator with poor conductivity. The band gap of silicene can also been tuned by substrates^11^,^12^,^13^ or surface adsorption^14^, where the high carrier mobility is kept. In addition, the half hydrogenated silicene on only one side exhibits ferromagnetic order^15^,^16^ while the half fluorinated silicene exhibits antiferromagnetic^17^, indicating that the patterning of functional atoms can tune the magnetic properties of silicene.

For practical applications, the two dimensional materials are usually cut into one dimensional nanoribbons. In addition, silicene nanoribbons (SNRs) offer the possibility to achieve tuneable band structures due to the size effect, i.e. the band gaps change with the width of the nanoribbons and also the orientation of edges. For example, the SNRs can be turned from semiconducting to metallic by manipulating these structural parameters^9^,^18^,^19^,^20^,^21^ similar to the case of graphene nanoribbons^22^,^23^. Unfortunately, manipulating the edge structure and width of freestanding SNRs are very challenging in experiments^18^,^19^. Alternatively, the high quality SNRs could be obtained by forming silicene/fluorosilicene hybrid nanoribbons (SFNRs) through patterning the fluorine atoms on the side rigions of silicene. The SFNRs might be fabricated by selectively fluorinating silicene by using a low-damage plasma treatment or exposing to atomic F formed by decomposition of XeF_2_ with masking techniques, or by removing F atoms from fluorosilicene by electron beam, as proposed for the graphene system^24^,^25^,^26^. However, both of experimental and theoretical studies on SFNRs are absent.

In this work, based on density functional theory (DFT) calculations, we will study the electronic and magnetic properties of the silicene/fluorosilicene hybrid nanoribbons. It has been reported that the energy barrier is proportional to the adsorption energy of adsorbates^27^. A large diffusion energy barrier indicates the high difficultly of F atoms moving, which reflects the stability of fluorinated silicene. In addition, the adsorbates are usually reactive at the interface. Therefore, the thermal stability of SFNRs is determined by calculating the diffusion barrier of F atoms at the silicene/fluorosilicene interface. All the possible diffusion pathways at the interface are analysed to search the minimum diffusion barrier, and therefore to provide guidance for designing viable silicene electronic devices that possess high thermal stability and excellent electronic and magnetic properties at the operating conditions.

## Results

**Figure 1:**
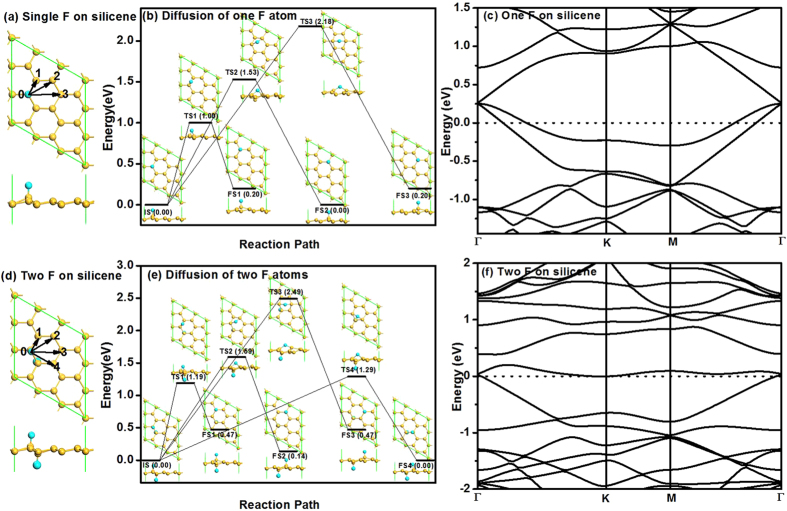
Atomic structure of silicene with one F atom (**a**) and two F atoms (**d**) after relaxation, where the arrows indicate the different diffusion pathways of F atoms. The numbers indicate different atomic positions. Panels (**b**,**e**) show the diffusion pathways of one F atom and two F atoms on pristine silicene, respectively. IS, TS and FS represent initial structure, transition structure and final structure, respectively. Their atomic structures are given by the inserts. The energy of IS is taken to be zero. The unit of *E*_bar_ and *E*_r_ is eV, where *E*_bar_ is the energy barrier and *E*_r_ is the reaction energy. The yellow and cyan atoms are respectively Si and F atoms in this and following figures. Panel (**c**,**f**) show the band structures of silicene with adsorption of one F atom and two F atoms, respectively. The dotted lines indicate the Fermi level.

We first investigate the diffusion of a single F atom on pristine silicene with 3 × 3 supercell as shown in Fig. 1(a). The buckled silicene has a buckling of Δ = 0.45 Å, which is similar to the literature data of 0.44 Å^9^. The F atom is chemically adsorbed on the Si atom at site 0 with formation energy of −3.63 eV based on eqn. (1). After the F atom binding, the Si atom at site 0 is lifted up 0.53 Å due to the *sp*^3^ hybrid. There are three possible reaction pathways for the diffusion of the F atom, i.e., from site 0 to site 1 (path 1), from site 0 to site 2 (path 2) and from site 0 to site 3 (path 3). As shown in Fig. 1(a), relative to the site 0 where the F atom adsorbed, the sites 1–3 denote the nearest Si, the second nearest Si, and opposite Si atoms, respectively. The Si atoms at sites 0 and 2 are on the upper layer of the buckled silicene, while those at site 1 and site 3 are on the lower layer of the buckled silicene. The configurations with the F atom adsorbed at sites 0–3 are initial state (IS), final state 1 (FS1), final state 2 (FS2) and final state 3 (FS3) during the transition state search, respectively. The detailed diffusion paths for a single F atom are calculated using LST/QST and NEB tools in Dmol^3^ and the results are shown in Fig. 1(b). It shows that the F atom diffusions along path 1 and path 3 are endothermic with energy barriers *E*_bar_ of 1.00 eV and 2.18 eV, respectively, while the energy barrier for the F atom diffusion along path 2 is 1.53 eV with zero reaction energy. Note that there is no energy difference before and after diffusion from site 0 to site 2 due to the equivalence of the 2 sites on the upper layer, while sites 1 and 3 are equivalent on the lower layer. In other words, FS1 and FS3 have the same total energy and it is 0.2 eV higher than IS and FS2. Therefore, the minimum diffusion energy barrier for single F atom on silicene is 1.00 eV along path 1. Based on the common criterion that a surface reaction at ambient temperature may occur when the energy barrier is smaller than the critical barrier of *E*_cbar_ = 0.91 eV^28^, the single F atom is considered to be stable on pristine silicene at room temperature. In order to study the effect of the simulation cell size on the results, we also studied the diffusion of single F atom on silicene with 4 × 4 and 5 × 5 supercells as shown in Figure S1. The minimum diffusion energy barriers for single F atom on silicene with 4 × 4 and 5 × 5 supercells are 1.05 and 1.06 eV along path 1, respectively, which is similar to the results obtained with the 3 × 3 supercell. Therefore, 3 × 3 supercell is used for the two dimensional silicene system in the following.

We also studied the diffusion of other halogen atom X (Cl, Br and I) on silicene for comparison purpose. Noted that the adsorption configurations and diffusion pathways for single Cl, Br and I atoms on silicene are similar to that of F atom, as shown in Figure S2 of supporting information. The minimum diffusion pathways are all along path 1, and the corresponding energy barriers for single Cl, Br and I atoms are 0.59, 0.45 and 0.35 eV, respectively. Since the minimum diffusion energy barriers are all smaller than the critical barrier of *E*_cbar_ = 0.91 eV^28^, the single Cl, Br and I atoms are not stable on pristine silicene, which does not satisfy the requirements of application in electronic devices. The different stability can be understood by calculating the formation energy of the halogen atoms on silicene, which are proportional to the strength of the Si–X bonds. DFT+D calculations show that the formation energies are −3.63, −1.65, −1.29 and −0.72 eV for F, Cl, Br and I adsorbed on silicene, respectively as shown in Table 1. The partial density of states (PDOS) for single F, Cl, Br and I atoms on silicene is shown in Figure S3 of supporting information, where more details for formation energy variation are given. As seen in Figures S3(a)–S3(d), the peaks of the Si−X band shift to the right from F to I, which implies that the Si−X interaction decreases and the adsorption of X atoms becomes weaker. This indicates that fluorinated silicene is the most stable, and thus we mainly focus on the diffusion of F atoms on silicene in the following.

The stability of two F atoms on pristine silicene is also considered. After careful examination, we find that the most favourable configuration is that two F atoms adsorbed on the two Si atoms next to each other on alternative side of silicene, as shown in Fig. 1(d). The formation energy is −3.75 eV for silicene with two F atoms on alternative side based on eqn (1), indicating enhanced stability with the presence of another F atom. There are four possible diffusion pathways for the *F* atom at site 0, i.e., from site 0 to site 1 (path 1), from site 0 to site 2 (path 2), from site 0 to site 3 (path3) and from site 0 to site 4 (path 4). The corresponding diffusion barriers are 1.19, 1.59, 2.49 and 1.29 eV for the pathways 1–4, respectively, and the minimum diffusion barrier is found to be 1.19 eV along path 1 as shown in Fig. 1(e). Therefore, with the presence of another F atom at the other side of silicene, the diffusion barrier increases remarkably from 1.00 to 1.19 eV, indicating the enhanced stability of F atoms on silicene at room temperature when the fluoridation rate of silicene is higher. Note that, similar to the diffusion of single F atom on silicene, FS1 and FS3 have the same energy since sites 1 and 3 are equivalent on the lower layer, while FS4 has the same energy as IS due to the equivalence of sites 0 and 4. For site 2, although it is on the upper layer similar to sites 0 and 4, the energy of FS2 is still different from those of IS and FS4 due to the presence of the second F atom on the other side of silicene. The band structures for silicene adsorbed with one and two F atoms are shown in Fig. 1(c,f), where the Fermi level crosses the energy bands, indicating that the metallic character is kept for both cases. When the silicene is fully fluorinated, the fluorosilicene turns to be semiconductor with a band gap of 1.20 eV, which is slightly smaller than the reported value of 1.469 due to the different exchange–correlation functional used^10^.

**Figure 2:**
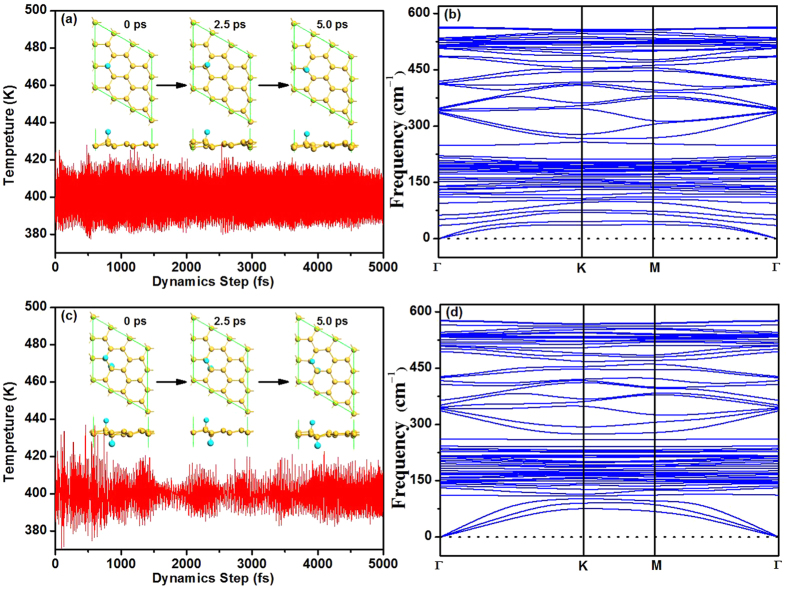
The dynamics process at *T* = 400 K in the NVT ensemble (a), and the phonon dispersion curve (b) for silicene with one F atom. Insets show three structures from MD calculations. Panel (**c**,**d**) show the dynamics process at *T* = 400 K in the NVT ensemble and the phonon dispersion curve for silicene with two F atoms, respectively.

To further investigate the stability of the silicene with one and two F atoms, ab initio molecular dynamics (MD) simulations at a constant temperature of *T* = 400 K in the NVT ensemble (i.e., constant particle number, volume and temperature condition) have been carried out for 5 ps with the time step of 1 fs. We selected this temperature (*T* = 400 K) because the work temperature for electronic device is usually higher than the room temperature. Three structures from MD calculation are present in Fig. 2(a,c), respectively. It is found that F atoms are located at the original sites after 5000 dynamics steps at 400 K. Thus, the stability of the studied silicene systems at room temperature is expected. We have also performed phonon calculations using the CASTEP code. Comparing the phonon dispersion curves shown in Fig. 2(b,d), we can see that there are no soft modes for the silicene adsorbed with F atoms, indicating that the studied structures correspond to local minimums. This is consistent with the fact that the fluorinated silicene is stable at room temperature when the diffusion energy barrier of F atoms on silicene is larger than the critical barrier of *E*_cbar_ = 0.91 eV^28^.

In addition, although the total energy of two F atoms adsorbed on the two Si atoms on alternative side of silicene is lower, the stability of two fluorine atoms at the same side on pristine silicene is also considered and it is found that the minimum direct diffusion barrier is 0.82 eV along path 1 as shown in Figure S4. With the presence of another F atom at the same side, the diffusion barrier decreases remarkably from 1.00 to 0.82 eV, indicating the weakened stability of F atoms on silicene. Therefore, the stability of F atoms on silicene can be enhanced through fluorinating both sides of silicene with stronger formation energy and higher diffusion energy barrier.

For comparison purpose, we also studied the diffusion of a F atom on graphene. Noted that the adsorption configurations and diffusion pathways for a single F and two F atoms on graphene are similar to those on silicene, as shown in Figure S5 of supporting information. The minimum diffusion energy barrier for single F atom on graphene is 0.40 eV along path 2, while the minimum diffusion barrier is found to be 0.93 eV along path 4 for two F atoms. Therefore, with the presence of another F atom at the other side of graphene, the stability of F atoms on graphene at room temperature is also enhanced.

**Figure 3:**
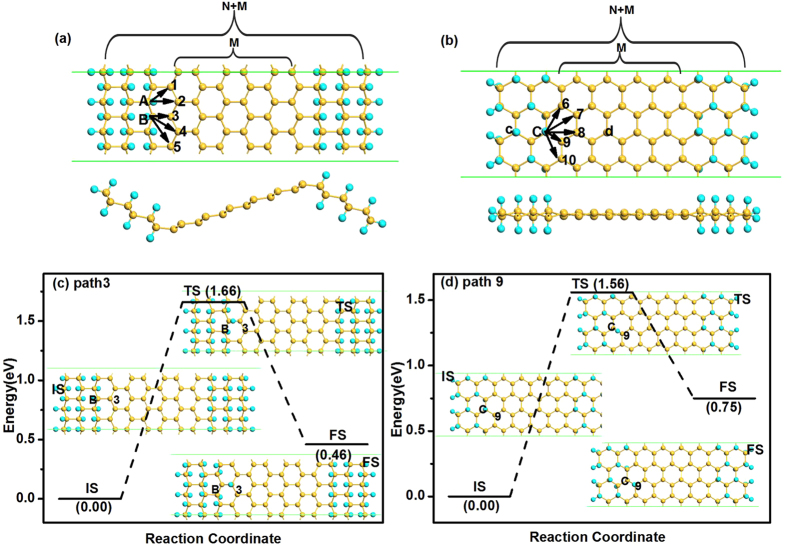
Atomic structure of zigzag 6/6-SFNR (**a**) and armchair 8/9-SFNR (**b**) after relaxation. The arrows indicate the different diffusion pathways of F atoms. The letters and numbers indicate different atomic positions. The diffusion pathway 3 of F atom on zigzag 6/6-SFNR (**c**), and the diffusion pathway 9 of F atom on armchair 8/9-SFNR (**d**).

Inspired by the stability enhancement of H atoms at silicene/silicane interface^29^, in this work we also consider the diffusion of F atoms at the silicene/fluorosilicene (SFNR) interface. The supercells used for the zigzag and armchair SFNRs are shown in Fig. 3(a,b), respectively. We minimized the interlayer interaction by allowing a vacuum width of 20 Å normal to the layer. For both types of nanoribbons, the buckling of fluorosilicene part increases to 0.68 Å due to the bonded F atoms. This value is similar to the buckling of Δ = 0.69 Å when F atoms adsorbed on silicene^10^. In both cases, this is a consequence of the change in the hybridization of the Si atoms from *sp*^2^ in silicene to *sp*^3^ in fluorosilicene. In addition, for the zigzag SFNR, both the silicene and the fluorosilicene nanoribbons are flat [see Fig. 3(a)]. However, the silicene and fluorosilicene layers are not in the same plane; they are connected with an angle of about 127° at the interface, which is similar to the previous reports for zigzag silicene/silicane nanoribbon^29^. For the armchair SFNR [see Fig. 3(b)], the silicene and fluorosilicene regions are almost in the same plane. As shown in Fig. 3, the width of the hybrid system is defined as *M* + *N* where *N* is the number of dimer lines (or zigzag chains) in fluorosilicene nanoribbon, and *M* is the number of dimer lines in centre silicene nanoribbon. In this work, two representative nanoribbons zigzag 6/6-SFNR and armchair 8/9-SFNR are studied in the following.

We now analyse the stability of the two types of interfaces by calculating the diffusion barriers for fluorine atoms at the interfaces. For the case of a zigzag interface (zigzag 6/6-SFNR), there are two types of Si and F atoms, denoted as sites A and B in Fig. 3(a). For the diffusion of the F atom at site A, there are two possible diffusion pathways labelled as 1 and 2 in Fig. 3(a). At the site B, there are three possible diffusion pathways for the F atom labelled as 3, 4, and 5. In the case of an armchair interface (armchair 8/9-SFNR), all the Si atoms at the interface are equivalent from a diffusion point of view. So there are five different diffusions pathways that we label as 6–10 in Fig. 3(b). When analysing the diffusion paths, we find that all the diffusions are along linear pathways.

The diffusion barriers of both types of silicene/fluorosilicene interfaces with different paths are summarized in Table 2. For the zigzag interface, the diffusion barriers at site A are 1.73 and 2.12 eV for the pathways 1 and 2, respectively, where the former is the minimum diffusion barrier at site A. The diffusion barriers at site B are 1.66, 2.79 and 2.48 eV for the pathways 3–5, respectively, where the minimum diffusion barrier at site B is 1.66 eV along path 3. As results, site A is slightly stable than site B, and the energy minimum diffusion pathway along the zigzag interface is pathway 3. After LST/QST and NEB calculations, the detailed reaction pathway and the energy barrier for the fluorine diffusion along the path 3 is shown in Fig. 3(c) where the initial state (IS), the final state (FS), and the atomic structure of transition state (TS) are given. For the armchair interface, the energy barriers at site C are respectively 1.75, 2.91, 2.74, 1.56 and 2.70 eV for diffusion pathways 6–10. Thus, the energy minimum diffusion path at armchair interfaces is path 9 from site C to the nearest Si atom with an energy barrier 1.56 eV. The corresponding reaction pathway and energy barrier are present in Fig. 3(d). Since the occurrence of surface diffusion needs *E*_bar_ < *E*_cbar_ = 0.91 eV at ambient temperature^28^, both of the zigzag and armchair interfaces are stable at room temperature. The formation energies are −4.13 and −4.16 eV for zigzag 6/6-SFNR and armchair 8/9-SFNR, which are much stronger than that of a single F atom on silicene, and thus further confirming the enhanced stability of SFNRs.

In light of the above analysis, we can see that the minimum diffusion barriers for both of armchair and zigzag interfaces are much larger than the energy barrier for the F diffusion on pristine silicene. From the diffusion energy in Table 2, the total energy increases ~ 0.5 eV after diffusion for all the cases. In Table 2, the reversing diffusion energy barrier *E′*_bar_ is also shown, which is much lower than the corresponding diffusion barrier *E*_bar._ Therefore, the exothermic reversing diffusion is energy preferable with lower diffusing energy barrier, which confirms the enhanced stability of the F atoms at silicene/fluorosilicene interfaces from another side. Note that *E′*_bar_ for F diffusion is defined as the energy difference between the final state and the TS state, and can be obtained from Table 2 as the difference between the diffusion barrier *E*_bar_ and the reaction energy *E*_r_.

Such stability enhancement can be understood by calculating the formation energy of the F atoms at different conditions, which are proportional to the strength of the Si–F bonds. For the zigzag interface, we found that the formation energy of the Si–F bond at sites A and B are −4.48 and −4.22, respectively. While for a F atom at site C of the armchair interface, this value is −4.19 eV. All of them are larger than the formation energy of an isolated F atom on a silicene supercell containing 18 Si atoms (−3.63 eV). This indicates the stability enhancement of the F atoms at silicene/fluorosilicene interfaces. The results of the formation energies also explain why it is easier to move the F atom from site B (*E*_f_ = −4.22 eV) than from site A (*E*_f_ = −4.48 eV) in the zigzag interface, and why moving the F atoms at site C (*E*_f_ = −4.19 eV) in the armchair interface is slightly easier than that at site B (*E*_f_ = −4.22 eV) in the zigzag interface. As results, the zigzag interface is slightly more stable than the armchair interface.

**Figure 4:**
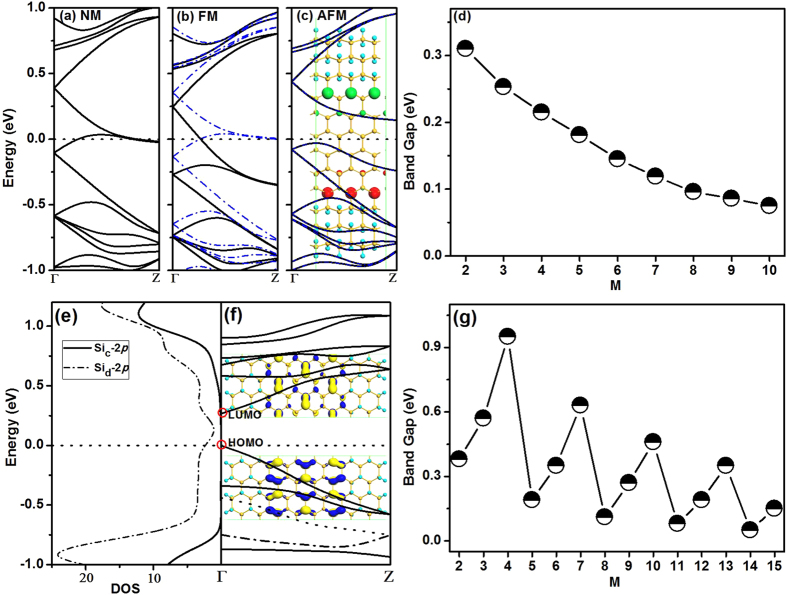
The band structures of zigzag 6/6-SFNR at (a) NM, (b) FM and (c) AFM states. Inset is the spin density distribution of zigzag 6/6-SFNR at AFM state, where the positive spin density is in red and the negative one is in green. (**d**) The M-dependence of band gaps of zigzag SFNRs with N + M = 12. The PDOS (**e**) and band structure (**f)** of armchair 8/9-SFNR. (**f**) The M-dependence of band gaps of armchair 8/9-SFNR with N + M = 17. The letters after the symbol of elements denote the atomic positions in the SFNRs. The dotted lines indicate the Fermi level. The charge distributions of LUMO and HOMO states at the Γ point are also given. The blue and yellow colours indicate different signs of orbital wave function.

To understand the electronic properties of SFNRs, the band structures of zigzag and armchair SFNRs are calculated and the results are shown in Fig. 4. For the zigzag edges, we have calculated the stabilities and electronic properties of the nonmagnetic NM state, ferromagnetic FM state, and antiferromagnetic AFM state. Figurs 4(a–c) show the band structures of zigzag 6/6-SFNR at different states. The NM and FM states are metallic, while the AFM state is semiconducting. According to our calculations, the relative total energies compared to AFM state are 15 and 63 meV for FM and NM states, respectively. Thus, similar to zigzag silicene nanoribbons^30^, the antiferromagnetic semiconductor is the ground state for zigzag SFNRs. The spin density distribution of AFM state is shown in Fig. 4(c), which exhibits that the spin states are originated from the hybrid *p*_z_ orbitals at the interface. In addition, the zigzag 6/6-SFNR has an indirect bandgap of about 0.14 eV. As the nanoribbon widths increase, the bandgaps of zigzag SFNRs decrease gradually. No oscillations appear in the hierarchy of zigzag SFNRs as shown in Fig. 4(d). The hydrogenated silicene nanoribbons have similar phenonminum when the width of the nanoribbon changes^30^.

For armchair 8/9-SFNR [see Fig. 4(f)], the Fermi level is on the exact top of the valence band and the armchair SFNRs changes to be a pristine semiconductor with a band gap of 0.27 eV and all the armchair SFNRs are nonmagnetic. The charge distribution of HOMO and LUMO states indicate that the band gap is mainly determined by the silicene nanoribbon. The partial density of states (PDOS) of the Si atom [site c in Fig. 3(b)] in the fluorosilicene part, and Si atom [site d in Fig. 3(b)] in the silicene part is shown in Fig. 4(e), where the conductive band minimum (CBM) and valence band maximum (VBM) are mostly contributed by Si−2*p* states in the silicene part. This is also consistent with the charge distribution of HOMO and LUMO states. In the armchair SFNRs, variations of the band gaps with M are summarized in Fig. 4(f) with N + M = 17. Clearly, the band gaps present oscillatory behavior and can be classified into three families with M = 3n, 3n + 1 and 3n + 2 (where n is a positive integer), respectively. The armchair SFNRs with M = 3n + 2 (M = 2, 5, 8, 11 and 14) have the smallest gaps, while the largest gaps correspond to M = 3n + 1 (M = 1, 4, 7, 10, 13 and 16). The hybrid system with M = 3n (M = 3, 6, 9, 12 and 15) has moderate band gaps than the former ones. The periodicity of the three families for band-gap oscillation is the same as that of armchair silicene nanoribbons^30^, consistent with that the electronic properties of the hybrid armchair system depend on the silicene part. We also tested the N-dependence of band gaps of armchair 8/9-SFNRs as displayed in Figure S6, and found that the energy gaps of N = 9 (0.26 eV) and 10 (0.26 eV) are nearly the same as N = 8 (0.27 eV), which confirms that the electronic properties of the hybrid armchair system depend on the silicene part not flourosilicene part. Therefore, the electronic properties of SFNRs can be tunned through controlling the width of silicene.

**Figure 5:**
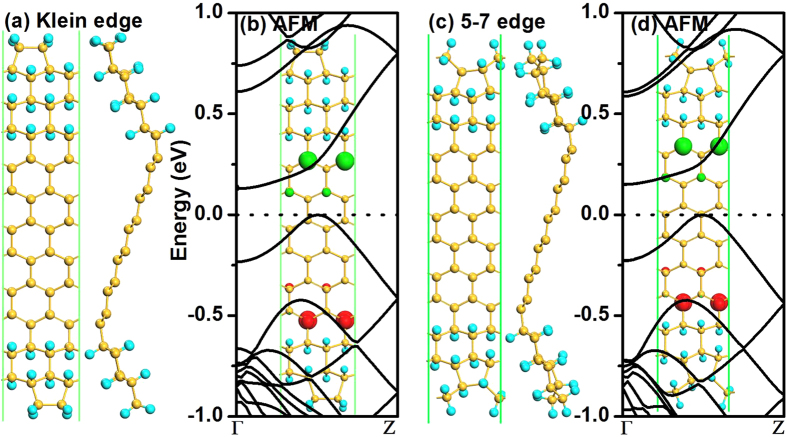
Atomic structure (**a**) and band structure at AFM state (**b**) of zigzag 6/6-SFNR with reconstructed Klein edge. Panels (**c**,**d**) show the atomic structure and band structure at AFM state of zigzag 6/6-SFNR with reconstructed 5–7 edge. Inset is the spin density distribution at AFM state, where the positive spin density is in red and the negative one is in green. The dotted lines indicate the Fermi level.

It is known that the edge reconstruction is very important to the structural stability and corresponding electronic properties. Actually, the experimenters have already observed two types of reconstructed edges for zigzag graphene nanoribbons, i.e., the Klein edge^31^ and the pentagon-heptagon (5–7) one^32^. These two kinds of edge reconstruction have also been studied for zigzag silicene nanoribbons^33^. Therefore, the Klein and 5–7 edge reconstructions for zigzag 6/6-SFNR have been studied as shown in Fig. 5. Similar to zigzag SFNRs, the antiferromagnetic semiconductor is the ground state with an indirect bandgap of about 0.14 eV for both cases, indicating that the hybrid SFNRs can protect the antiferromagnetic semiconducting character from the edge reconstructions. The formation energies are −4.09 and −4.10 eV for zigzag 6/6-SFNR with Klein and 5−7 reconstructions, respectively, confirming the stability of reconstructed SFNR.

## Discussion

In summary, we have studied the stability of SFNRs with both zigzag and armchair interfaces by calculating the diffusion barriers of F atoms using DFT method. We found that the stability of F atoms at the silicene/fluorosilicene interfaces is enhanced significantly, compared with the case of an isolated fluorine atom and a pair of fluorine atoms on pristine silicene. This enhancement is induced by the increase of the Si–F bond strength at the silicene/silicane interfaces. In addition, the armchair SFNRs are nonmagnetic semiconductors and the band gap presents oscillatory behavior when the width of silicene part changing. For the zigzag interfaces, we find that the antiferromagnetic semiconducting state is the ground state of zigzag SFNRs. Our results show that both types of silicene/fluorosilicene interfaces in hybrid nanoribbons are rather stable, which increases the feasibility for future technological applications of these systems. In addition, the electronic properties of SFNRs can be tuned through controlling the width of the silicene ribbon.

## Methods

The spin−unrestricted DFT calculations are carried out by using Dmol^3^ package^34^. Generalized gradient approximation (GGA) with Perdew−Burke− Ernzerhof (PBE)^35^ is taken as the exchange−correlation function. DFT semicore pseudopotentials (DSPPs) core treatment is implemented for relativistic effects, which replaces core electrons by a single effective potential. Double numerical plus polarization (DNP) is employed as the basis set. The convergence tolerance of energy of 10^−5^ Hartree is taken (1 Hartree = 27.21 eV), and the maximal allowed force and displacement are 0.002 Hartree/Å and 0.005 Å, respectively. It was reported that the selection of exchange−correlation functional has evidential effect on the result of adsorption energies. However, the effect on the calculated reaction energy barriers is much smaller^36^. To investigate the minimum energy pathway for the diffusion of H atoms at the silicene/silicane interface, linear synchronous transit/quadratic synchronous transit (LST/QST)^37^ and nudged elastic band (NEB)^38^ tools in Dmol^3^ module are used, which have been well validated to determine the structure of the transition state and the minimum energy reaction pathway. The DFT+D method within the Grimme scheme^39^ is used in all calculations to consider the van der Waals forces. In the simulation, three−dimensional periodic boundary conditions are taken. The simulation cell for pristine silicene consists of a 3 × 3 silicene supercell with a vacuum width of 20 Å above the silicene layer to minimize the interlayer interaction, where the *k*−point is set to 6 × 6 × 1, and all atoms are allowed to relax according to previous reports^40^. For the nanoribbons, the *k*−point is set to 1 × 1 × 6 along the periodic direction after careful examinations. The Phonon dispersions of the silicene are calculated by using CASTEP code^41^, where the ultrasoft pseudopotentials, finite displacement method, the GGA-PBE functional, an energy cutoff of 400 eV and 12 × 12 × 1 k-point meshes are used.

To evaluate the structural stability of F-silicenes (or SFNRs), the formation energy *E*_f_ for the silicene system is listed in Table 1 and Table 2, where *E*_f_ is defined as^17^,


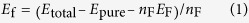


where *E*_total_ is the total energy of the F-silicene (or SFNRs), *E*_pure_ is that of pristine silicene (or SFNRs), *E*_F_ is taken as the energy of per F atom of an F_2_ molecule, and *n*_F_ denote the number of F atoms in the corresponding systems.

## Additional Information

**How to cite this article**: Jiang, Q. G. *et al.* First Principles Study on the Electronic Structure and Interface Stability of Hybrid Silicene/Fluorosilicene Nanoribbons. *Sci. Rep.* **5**, 15734; doi: 10.1038/srep15734 (2015).

## Supplementary Material

Supplementary Information

## Figures and Tables

**Table 1:** Structural and electronic properties of X-silicenes.

Structure		*l*_Si-X_	*q*(Si)	*q*(X)	*E*_f_	*E*_bar_	
F	single	1.642	0.484	−0.428	−3.63	1.00	
	two	1.643	0.447	−0.435	−3.75	1.19	
Cl	single	2.114	0.167	−0.300	−1.65	0.59	
Br	single	2.309	0.302	−0.307	−1.29	0.45	
I	single	2.553	0.201	−0.307	−0.72	0.35	

*l*_Si–X_ (Å), *q*(Si), *q*(X), *E*_f_ (eV) and *E*_bar_ (eV) stand for Si–X bond lengths, Mulliken charge of Si atom and X atom binding with X atom, formation energy *E*_f_ and minimum diffusion energy barrier for X atoms on silicene.

**Table 2:** Energy barrier *E*_bar_ and diffusion energy *E*_r_ for several diffusion paths on zigzag 6/6-SFNR and armchair 8/9-SFNR as indicated in Fig. 3.

Diffusion pathway			*E*_bar_	*E*_r_	*E′*_bar_	*E*_f_
Zigzag	A	1	1.73	0.63	0.99	−4.13
		2	2.12	0.94	0.65	
	B	**3**	**1.66**	0.46	1.20	
		4	2.79	0.62	2.17	
		5	2.48	0.39	2.09	
Armchair	C	6	1.75	0.49	1.26	−4.16
		7	2.91	0.82	2.09	
		8	2.74	0.50	2.24	
		**9**	**1.56**	0.75	0.81	
		10	2.70	0.23	2.47	

The energy barrier *E′*_bar_ for reversing diffusion of a F atom is also shown. A, B, C and the numbers from 1 to 10 indicate different atomic positions as shown in Fig. 3. Formation energy *E*_f_ for zigzag 6/6-SFNR and armchair 8/9-SFNR is also given.
