# Supplementary material for: First Principles Study on the Electronic Structure and Interface Stability of Hybrid Silicene/Fluorosilicene Nanoribbons
Source: Sci Rep. 2015 Oct 26;5:15734. doi: 10.1038/srep15734 (PMC4620566; doi:10.1038/srep15734)
Supplement: Supplementary Information [file srep15734-s1.doc]

**Supporting Information**

*for*

**First Principles Study on the Electronic Structure and Interface Stability of Hybrid Silicene/Fluorosilicene Nanoribbons**

Q. G. Jiang,^1^ J. F. Zhang,1[[1]](#footnote-2) Z. M. Ao^2*^, Y. P. Wu^1^

1 College of Mechanics and Materials, Hohai University, Nanjing 210098, China

2 Centre for Clean Energy Technology, School of Mathematical and Physical Sciences, University of Technology Sydney, PO Box 123, Broadway, Sydney, NSW 2007, Australia


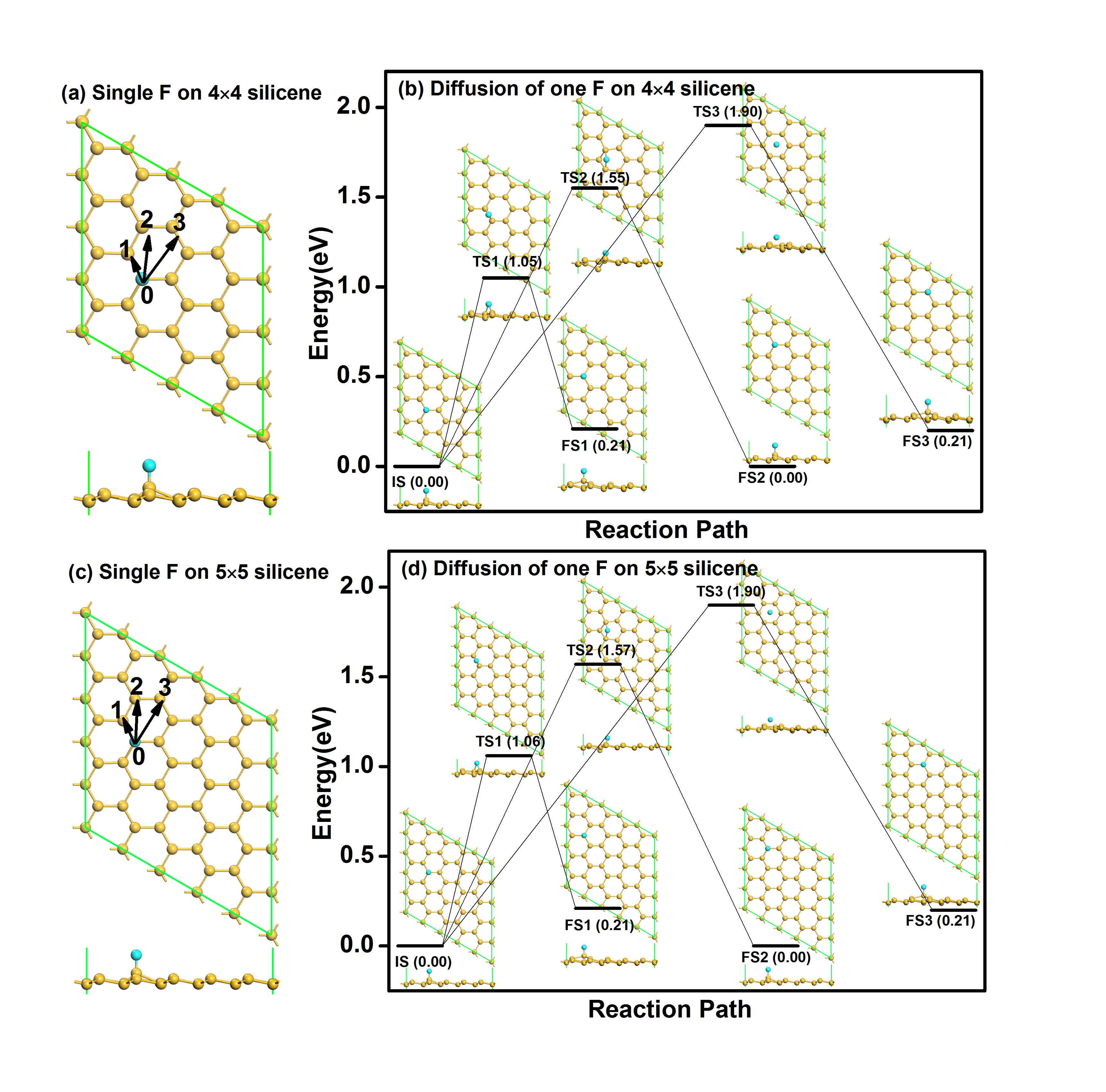


**Figure S1 ** Atomic structure of silicene with one F atom on silicene with 4 × 4 supercell (a) and 5 × 5 supercell (c), where the arrows indicate the different diffusion pathways of F atom. The numbers indicate different atomic positions. Panels (b) and (d) show the diffusion pathways of one F atom and two F atoms on pristine silicene, respectively. IS, TS and FS represent initial structure, transition structure and final structure, respectively. Their atomic structures are given by the inserts. The energy of IS is taken to be zero. The unit of *E*_bar_ and *E*_r_ is eV, where *E*_bar_ is the energy barrier and *E*_r_ is the reaction energy. The yellow and cyan atoms are respectively Si and F atoms in this and following figures.


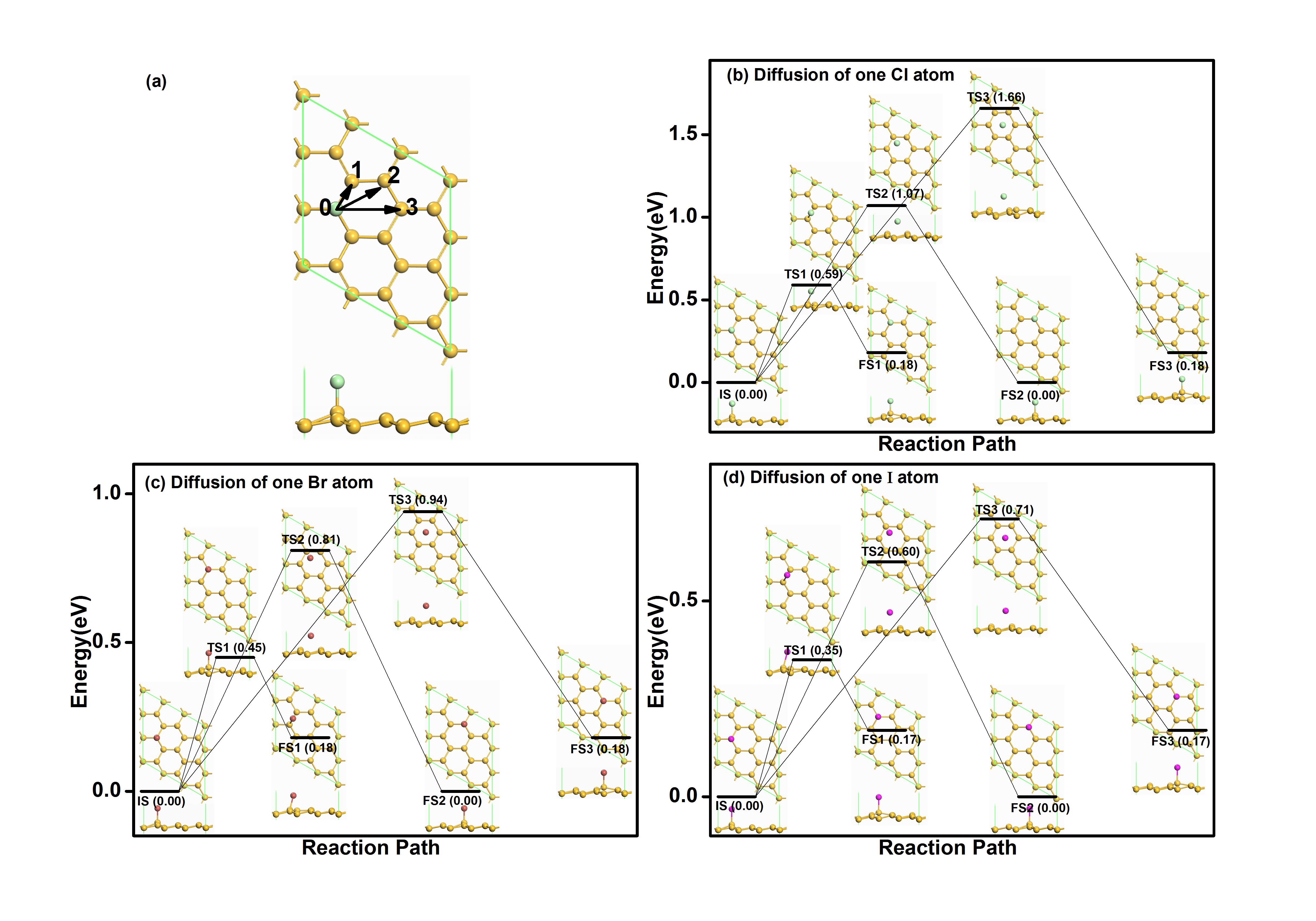


**Figure S2 ** Atomic structure of pristine silicene with one Cl atom (a) after relaxation, where the arrows indicate the different diffusion pathways of Cl atoms. The numbers indicate different atomic positions. Panels (b)-(d) show the diffusion pathways of one Cl, Br and I atoms on pristine silicene, respectively.


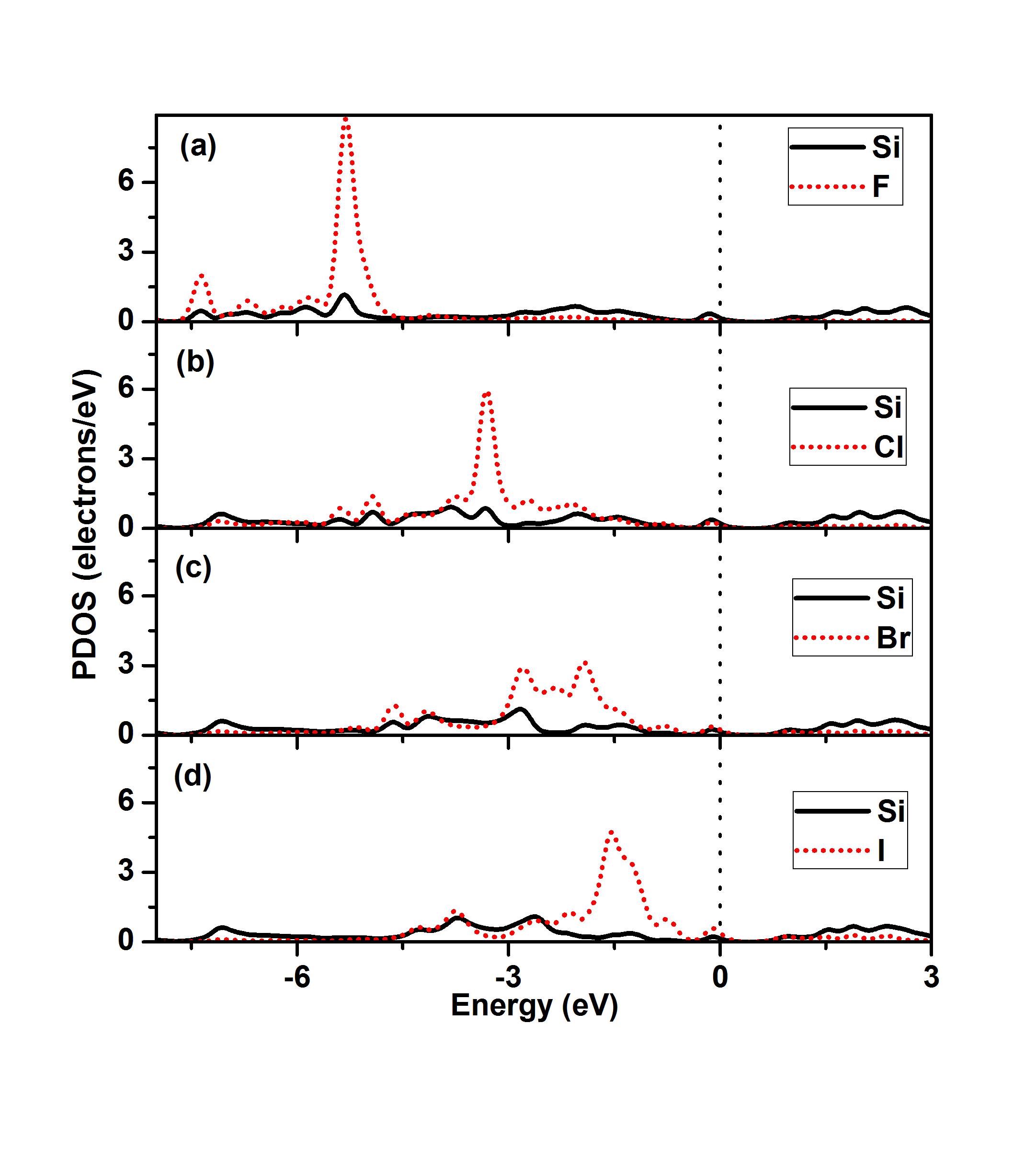


**Figure S3 ** Panels (a)-(d) show the PDOS of F, Cl, Br and I (dotted line) and Si (solid line) at the 0 site for one F, Cl, Br and I atoms on pristine silicene, respectively. The Fermi level is labelled as a dotted line.


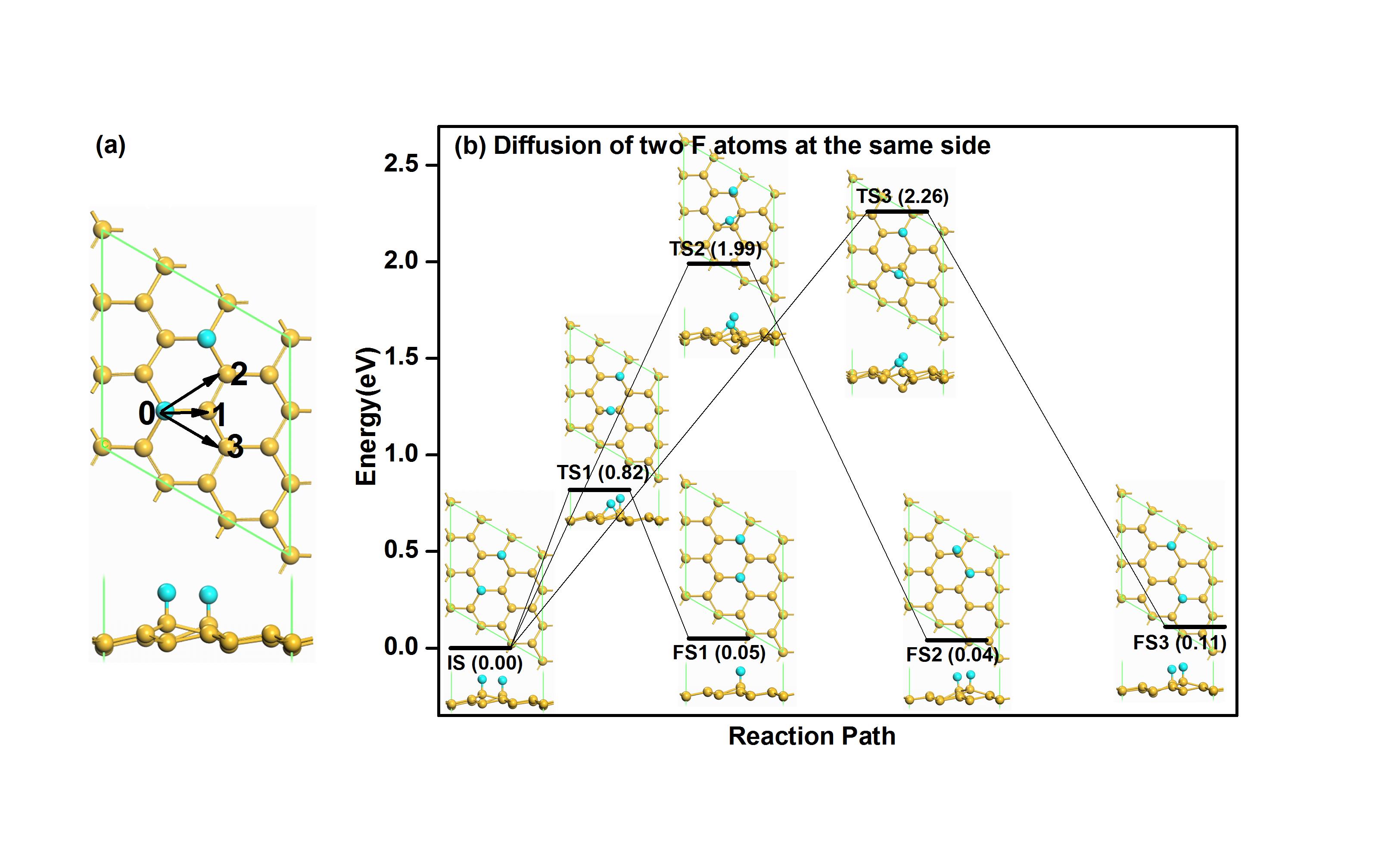


**Figure S4 ** Atomic structure of pristine silicene adsorbed with two F atoms at the same side (a) after relaxation, where the arrows indicate the different diffusion pathways of F atoms at site 0. Panels (b) shows the diffusion pathways of two F atoms adsorbed at the same side on pristine silicene. The energy of IS is taken to be zero.


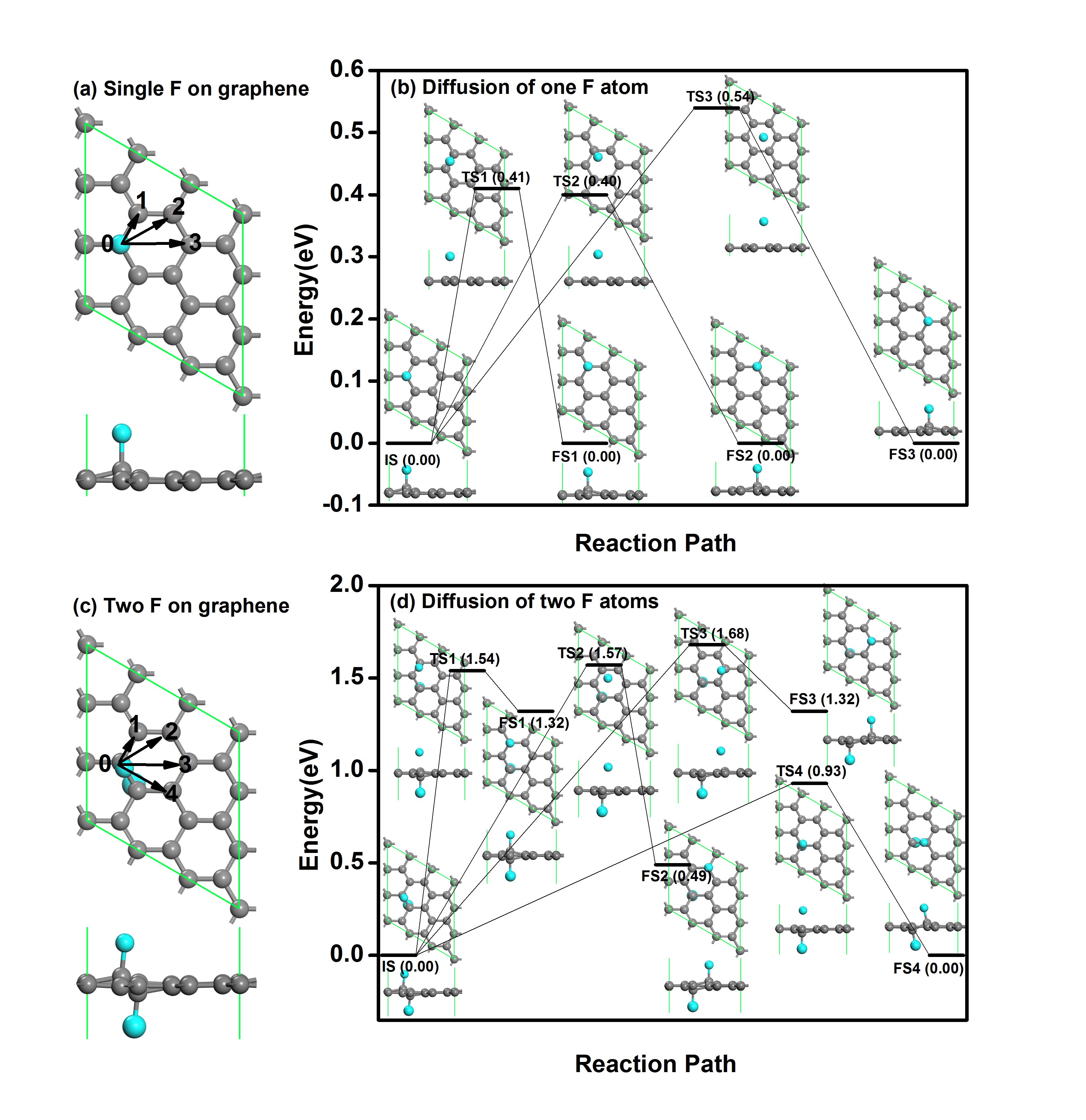


**Figure S5 ** Atomic structure of graphene with one F atom (a) and two F atoms (c) after relaxation, where the arrows indicate the different diffusion pathways of F atoms. The numbers indicate different atomic positions. Panels (b) and (d) show the diffusion pathways of one F atom and two F atoms on pristine graphene, respectively. The gray and cyan atoms are respectively C and F atoms.


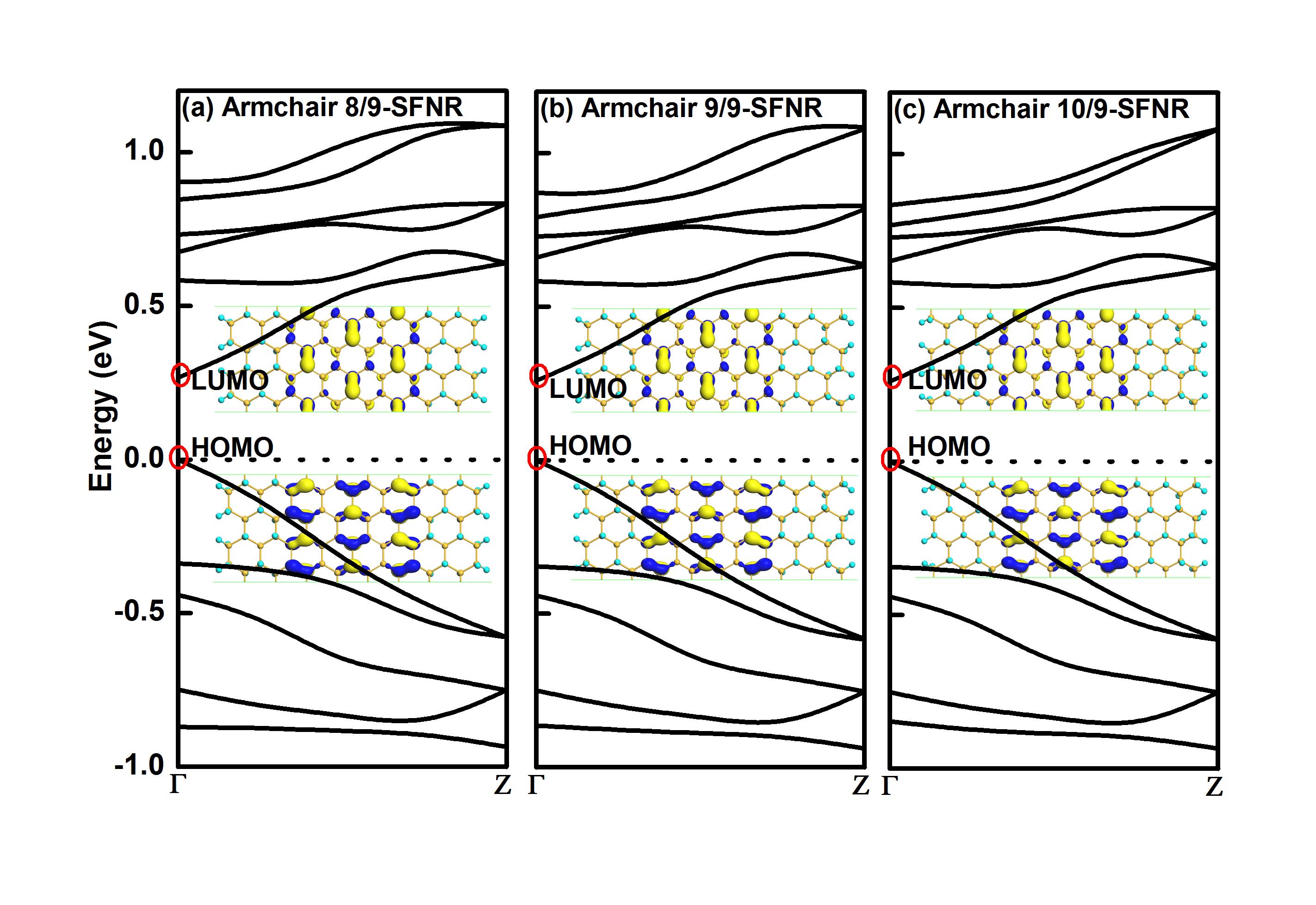


**Figure S6 ** The band structures of armchair 8/9-SFNR (a), 9/9-SFNR (b), and 10/9-SFNR (c). The dotted lines indicate the Fermi level. The charge distributions of LUMO and HOMO states at the Γ point are also given. The blue and yellow colours indicate different signs of orbital wave function.

1. Corresponding authors: [jfzhang@hhu.edu.cn](mailto:jfzhang@hhu.edu.cn); zhimin.ao@uts.edu.au [↑](#footnote-ref-2)
